# Supplementary material for: Pyruvate Dehydrogenase Kinase 1 inhibition mediated oxidative phosphorylation enhancement in cartilage promotes osteoarthritis progression
Source: BMC Musculoskelet Disord. 2023 Jul 20;24:597. doi: 10.1186/s12891-023-06585-6 (PMC10357736; doi:10.1186/s12891-023-06585-6)
Supplement: Supplementary file 1 — Additional file 1. [file 12891_2023_6585_MOESM1_ESM.zip › KEGG.Gsea.1653622432464/KEGG_LYSINE_DEGRADATION.html]

Details for gene set KEGG\_LYSINE\_DEGRADATION[GSEA]

|  || Dataset | 55457\_collapsed\_to\_symbols.cls.cls #OA\_versus\_normal.cls.cls #OA\_versus\_normal\_repos |
| Phenotype | cls.cls#OA\_versus\_normal\_repos |
| Upregulated in class | normal |
| GeneSet | KEGG\_LYSINE\_DEGRADATION |
| Enrichment Score (ES) | -0.26204425 |
| Normalized Enrichment Score (NES) | -0.953627 |
| Nominal p-value | 0.53109246 |
| FDR q-value | 0.72075033 |
| FWER p-Value | 1.0 |
Table: GSEA Results Summary

  

Fig 1: Enrichment plot: KEGG\_LYSINE\_DEGRADATION      
 Profile of the Running ES Score & Positions of GeneSet Members on the Rank Ordered List

  

| SYMBOL | TITLE | RANK IN GENE LIST | RANK METRIC SCORE | RUNNING ES | CORE ENRICHMENT || 1 | ALDH2 | aldehyde dehydrogenase 2 family member [Source:HGNC Symbol;Acc:HGNC:404] | 1003 | 0.517 | -0.0266 | No |
| 2 | ACAT1 | acetyl-CoA acetyltransferase 1 [Source:HGNC Symbol;Acc:HGNC:93] | 1017 | 0.513 | 0.0233 | No |
| 3 | ALDH3A2 | aldehyde dehydrogenase 3 family member A2 [Source:HGNC Symbol;Acc:HGNC:403] | 1263 | 0.465 | 0.0505 | No |
| 4 | ECHS1 | "enoyl-CoA hydratase, short chain 1 [Source:HGNC Symbol;Acc:HGNC:3151]" | 1925 | 0.369 | 0.0358 | No |
| 5 | ALDH7A1 | aldehyde dehydrogenase 7 family member A1 [Source:HGNC Symbol;Acc:HGNC:877] | 2158 | 0.343 | 0.0518 | No |
| 6 | ALDH9A1 | aldehyde dehydrogenase 9 family member A1 [Source:HGNC Symbol;Acc:HGNC:412] | 2795 | 0.270 | 0.0292 | No |
| 7 | PLOD1 | "procollagen-lysine,2-oxoglutarate 5-dioxygenase 1 [Source:HGNC Symbol;Acc:HGNC:9081]" | 3155 | 0.235 | 0.0246 | No |
| 8 | SETMAR | SET domain and mariner transposase fusion gene [Source:HGNC Symbol;Acc:HGNC:10762] | 3216 | 0.229 | 0.0427 | No |
| 9 | PLOD3 | "procollagen-lysine,2-oxoglutarate 5-dioxygenase 3 [Source:HGNC Symbol;Acc:HGNC:9083]" | 3614 | 0.192 | 0.0309 | No |
| 10 | HADHA | hydroxyacyl-CoA dehydrogenase trifunctional multienzyme complex subunit alpha [Source:HGNC Symbol;Acc:HGNC:4801] | 4163 | 0.141 | 0.0024 | No |
| 11 | SETDB1 | SET domain bifurcated histone lysine methyltransferase 1 [Source:HGNC Symbol;Acc:HGNC:10761] | 4891 | 0.080 | -0.0462 | No |
| 12 | AASDHPPT | aminoadipate-semialdehyde dehydrogenase-phosphopantetheinyl transferase [Source:HGNC Symbol;Acc:HGNC:14235] | 5047 | 0.069 | -0.0514 | No |
| 13 | HADH | hydroxyacyl-CoA dehydrogenase [Source:HGNC Symbol;Acc:HGNC:4799] | 5088 | 0.065 | -0.0480 | No |
| 14 | NSD1 | nuclear receptor binding SET domain protein 1 [Source:HGNC Symbol;Acc:HGNC:14234] | 5178 | 0.058 | -0.0492 | No |
| 15 | EHMT2 | euchromatic histone lysine methyltransferase 2 [Source:HGNC Symbol;Acc:HGNC:14129] | 5507 | 0.032 | -0.0715 | No |
| 16 | SUV39H1 | suppressor of variegation 3-9 homolog 1 [Source:HGNC Symbol;Acc:HGNC:11479] | 6028 | -0.007 | -0.1113 | No |
| 17 | KMT5B | lysine methyltransferase 5B [Source:HGNC Symbol;Acc:HGNC:24283] | 6198 | -0.020 | -0.1224 | No |
| 18 | NSD3 | nuclear receptor binding SET domain protein 3 [Source:HGNC Symbol;Acc:HGNC:12767] | 6220 | -0.022 | -0.1219 | No |
| 19 | ACAT2 | acetyl-CoA acetyltransferase 2 [Source:HGNC Symbol;Acc:HGNC:94] | 6291 | -0.027 | -0.1247 | No |
| 20 | ALDH1B1 | aldehyde dehydrogenase 1 family member B1 [Source:HGNC Symbol;Acc:HGNC:407] | 6551 | -0.046 | -0.1403 | No |
| 21 | PIPOX | pipecolic acid and sarcosine oxidase [Source:HGNC Symbol;Acc:HGNC:17804] | 6781 | -0.061 | -0.1520 | No |
| 22 | SETD2 | "SET domain containing 2, histone lysine methyltransferase [Source:HGNC Symbol;Acc:HGNC:18420]" | 6926 | -0.074 | -0.1558 | No |
| 23 | NSD2 | nuclear receptor binding SET domain protein 2 [Source:HGNC Symbol;Acc:HGNC:12766] | 7622 | -0.129 | -0.1971 | No |
| 24 | EHHADH | enoyl-CoA hydratase and 3-hydroxyacyl CoA dehydrogenase [Source:HGNC Symbol;Acc:HGNC:3247] | 7968 | -0.153 | -0.2087 | No |
| 25 | GCDH | glutaryl-CoA dehydrogenase [Source:HGNC Symbol;Acc:HGNC:4189] | 8180 | -0.170 | -0.2083 | No |
| 26 | OGDH | oxoglutarate dehydrogenase [Source:HGNC Symbol;Acc:HGNC:8124] | 8503 | -0.194 | -0.2141 | No |
| 27 | SUV39H2 | suppressor of variegation 3-9 homolog 2 [Source:HGNC Symbol;Acc:HGNC:17287] | 8529 | -0.196 | -0.1965 | No |
| 28 | TMLHE | "trimethyllysine hydroxylase, epsilon [Source:HGNC Symbol;Acc:HGNC:18308]" | 8796 | -0.216 | -0.1957 | No |
| 29 | BBOX1 | gamma-butyrobetaine hydroxylase 1 [Source:HGNC Symbol;Acc:HGNC:964] | 8814 | -0.218 | -0.1754 | No |
| 30 | DOT1L | DOT1 like histone lysine methyltransferase [Source:HGNC Symbol;Acc:HGNC:24948] | 8832 | -0.220 | -0.1549 | No |
| 31 | PLOD2 | "procollagen-lysine,2-oxoglutarate 5-dioxygenase 2 [Source:HGNC Symbol;Acc:HGNC:9082]" | 10086 | -0.330 | -0.2195 | No |
| 32 | DLST | dihydrolipoamide S-succinyltransferase [Source:HGNC Symbol;Acc:HGNC:2911] | 10634 | -0.386 | -0.2237 | Yes |
| 33 | OGDHL | oxoglutarate dehydrogenase L [Source:HGNC Symbol;Acc:HGNC:25590] | 10754 | -0.399 | -0.1933 | Yes |
| 34 | SETD1B | "SET domain containing 1B, histone lysine methyltransferase [Source:HGNC Symbol;Acc:HGNC:29187]" | 11235 | -0.461 | -0.1848 | Yes |
| 35 | EHMT1 | euchromatic histone lysine methyltransferase 1 [Source:HGNC Symbol;Acc:HGNC:24650] | 11686 | -0.530 | -0.1671 | Yes |
| 36 | ASH1L | ASH1 like histone lysine methyltransferase [Source:HGNC Symbol;Acc:HGNC:19088] | 12405 | -0.708 | -0.1526 | Yes |
| 37 | SETD1A | "SET domain containing 1A, histone lysine methyltransferase [Source:HGNC Symbol;Acc:HGNC:29010]" | 12793 | -0.954 | -0.0880 | Yes |
| 38 | AASS | aminoadipate-semialdehyde synthase [Source:HGNC Symbol;Acc:HGNC:17366] | 12799 | -0.968 | 0.0079 | Yes |
Table: GSEA details [plain text format]

  

Fig 2: KEGG\_LYSINE\_DEGRADATION      
 Blue-Pink O' Gram in the Space of the Analyzed GeneSet

  

Fig 3: KEGG\_LYSINE\_DEGRADATION: Random ES distribution      
 Gene set null distribution of ES for **KEGG\_LYSINE\_DEGRADATION**
